# Automated model optimization to study spike shape modulation in Layer 2/3 cortical pyramidal neurons

**DOI:** 10.1186/1471-2202-13-S1-P57

**Published:** 2012-07-16

**Authors:** Michael Vella, Hugh PC Robinson

**Affiliations:** 1 Department of Physiology,Development and Neuroscience, University of Cambridge, Cambridge, CB2 3DY, UK

## 

It has been proposed that, contrary to the “classical” view in which cortical action potentials are encoded as purely digital, all-or-none events, action potential (AP) shape may be used by the brain for representing and processing information [1-4]. Cortical pyramidal neurons of Layer 2/3 show prominent variations in AP waveform during sustained depolarizing responses, which may lead to different levels of synaptic output at proximal axonal synaptic terminals, and different patterns of invasion of the dendrites by back-propagating APs. A range of membrane ionic channels probably plays an important part in this phenomenon. We carried out electrical recording during conductance injection, combined with morphological reconstruction and multicompartmental modelling of Layer II/III pyramidal neurons in rat and mouse cortical slices, to investigate the mechanism of AP waveform modulation. Evolutionary optimization techniques [5-7] were implemented and a computational cluster architecture was designed for searching the parameter space of ion channel distributions and properties efficiently in parallel, to fit models to experimental data. As a test of the fitting process, we assessed its ability to detect point voltage-dependent conductances of different types, introduced at the soma by conductance injection (dynamic clamp). Using the available evidence, we describe how the inactivation kinetics of voltage-dependent potassium channels appears to play a particularly important role in the modulation of action potential waveform of these neurons.
